# A possible role for fumagillin in cellular damage during host infection by *Aspergillus fumigatus*

**DOI:** 10.1080/21505594.2018.1526528

**Published:** 2018-09-25

**Authors:** Xabier Guruceaga, Guillermo Ezpeleta, Emilio Mayayo, Monica Sueiro-Olivares, Ana Abad-Diaz-De-Cerio, José Manuel Aguirre Urízar, Hong G. Liu, Philipp Wiemann, Jin Woo Bok, Scott G. Filler, Nancy P. Keller, Fernando L. Hernando, Andoni Ramirez-Garcia, Aitor Rementeria

**Affiliations:** a Fungal and Bacterial Biomics Research Group, Department of Immunology, Microbiology and Parasitology, Faculty of Science and Technology, University of the Basque Country (UPV/EHU), Leioa, Spain; b Preventive Medicine and Hospital Hygiene Service, Complejo Hospitalario de Navarra, Pamplona, Spain; c Department of Preventive Medicine and Public Health, Faculty of Medicine and Nursing, University of the Basque Country (UPV/EHU), Leioa, Spain; d Pathology Unit, Medicine and Health Science Faculty, University of Rovira i Virgili, Reus, Tarragona, Spain; e Department of Stomatology II, Faculty of Medicine and Nursing, University of the Basque Country (UPV/EHU), Leioa, Spain; f Division of Infectious Diseases, Los Angeles Biomedical Research Institute at Harbor-UCLA Medical Center, Torrance, CA, USA; g Department of Medicine, David Geffen School of Medicine at UCLA, Los Angeles, CA, USA; h Department of Medical Microbiology and Immunology, University of Wisconsin, Madison, WI, USA; i Department of Bacteriology, University of Wisconsin, Madison, WI, USA

**Keywords:** *Aspergillus*, fumagillin, intranasal infection, AWAFUGE, epithelial cells, fumagillin, cytotoxicity, virulence

## Abstract

Virulence mechanisms of the pathogenic fungus *Aspergillus fumigatus* are multifactorial and depend on the immune state of the host, but little is known about the fungal mechanism that develops during the process of lung invasion. In this study, microarray technology was combined with a histopathology evaluation of infected lungs so that the invasion strategy followed by the fungus could be described. To achieve this, an intranasal mice infection was performed to extract daily fungal samples from the infected lungs over four days post-infection. The pathological study revealed a heavy fungal progression throughout the lung, reaching the blood vessels on the third day after exposure and causing tissue necrosis. One percent of the fungal genome followed a differential expression pattern during this process. Strikingly, most of the genes of the intertwined fumagillin/pseurotin biosynthetic gene cluster were upregulated as were genes encoding lytic enzymes such as lipases, proteases (DppIV, DppV, Asp f 1 or Asp f 5) and chitinase (chiB1) as well as three genes related with pyomelanin biosynthesis process. Furthermore, we demonstrate that fumagillin is produced in an *in vitro* pneumocyte cell line infection model and that loss of fumagillin synthesis reduces epithelial cell damage. These results suggest that fumagillin contributes to tissue damage during invasive aspergillosis. Therefore, it is probable that *A. fumigatus* progresses through the lungs via the production of the mycotoxin fumagillin combined with the secretion of lytic enzymes that allow fungal growth, angioinvasion and the disruption of the lung parenchymal structure.

## Introduction

*Aspergillus fumigatus* is a filamentous fungus with a worldwide distribution that fulfills an important environmental role degrading organic matter in decomposition [1]. However, it has also become the most serious etiological agent of invasive mold infections [2,3]. The small size of its conidia not only allows it to spread through the air [4], but also allows them to reach deep into the human respiratory system [5]. Indeed, the lung is the main target organ and is where the fungus can cause a broad spectrum of disease, ranging from allergic responses to invasive pulmonary aspergillosis. In the bronchial tree, the combined action of the ciliated epithelium [6] and the alveolar immune cells [7] attempt to eliminate any infection, but the specific mechanisms for conidia recognition and removal are incompletely understood [8].

Fungal virulence mechanisms involved in development of infection are multifactorial, some of them being necessary to invade tissues and evade the immune response [9–12]. During the host-pathogen interaction, *A. fumigatus* undergoes changes in its protein expression patterns, where studies have identified up-regulated proteins including allergens [13], immunogenic proteins [14], new putative diagnostic targets [15] and toxins/secondary metabolites [16] among others. In addition to these findings, studies of the changes in gene expression during encounters with host tissues or in stress conditions have presented genes encoding proteins possibly involved in virulence [17–20]. However, up to date, only a few experiments have been performed using organisms isolated from bronchoalveolar lavage from intranasal infection models [21,22].

It is known that a large number of modifications in the gene expression pattern are observed during the first few hours after conidia break their dormancy, most of these genes being related to fungal growth and metabolism, but not specifically to virulence [23]. In consequence, the aim of this study was to describe the modifications in gene expression of *A. fumigatus* during pulmonary infection once conidia have settled in the lung. To perform this study, a microarray analysis of *A. fumigatus* gene expression during a murine intranasal infection model was performed. Transcriptomic profiles from early (24 h) and advanced (72 and 96 h) stages of infection were compared to identify gene expression patterns, which were verified by RT-qPCR. Notably, 14 genes of the 21 intertwined fumagillin/pseurotin gene cluster [24] were found to be upregulated at the advanced time points. Furthermore, an *A. fumigatus ∆fmaA* (fumagillin null) mutant caused significantly less damage that the wild-type strain to a pulmonary epithelial cell line *in vitro*, suggesting a role for fumagillin in inducing cellular injury.

## Results

### Survival of animals after *aspergillus* infection, selection of doses and progression of fungal burden in the lung

**Figure 1.**
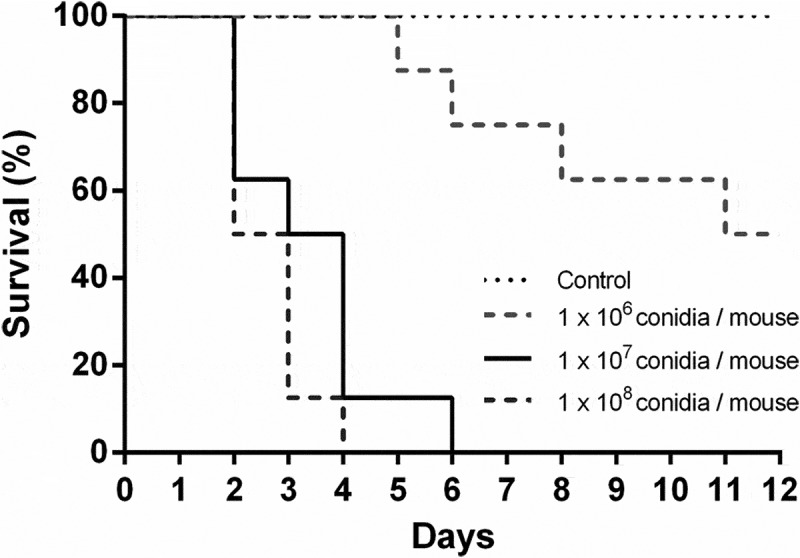
**Survival analysis of immunosuppressed mice intranasally infected with *Aspergillus fumigatus***. Mice were immunosuppressed with two intraperitoneal doses of cyclophosphamide, 150 mg/Kg and 100 mg/Kg, four days and one day before infection, respectively. Animals were infected with 1 x 10^6^, 1 × 10^7^ and 1 × 10^8^ *A. fumigatus* resting conidia/animal. Non-infected animals were used as controls.

Three doses of *A. fumigatus* conidia were tested in a leukopenic mouse model of invasive aspergillosis to find the most appropriate one for transcriptomic studies. Figure 1 summarizes the survival results obtained with each dose of conidia used. The selected infective dosage was 1 × 10^7^ conidia per mouse, as it resulted in a 90% mortality.

**Figure 2.**
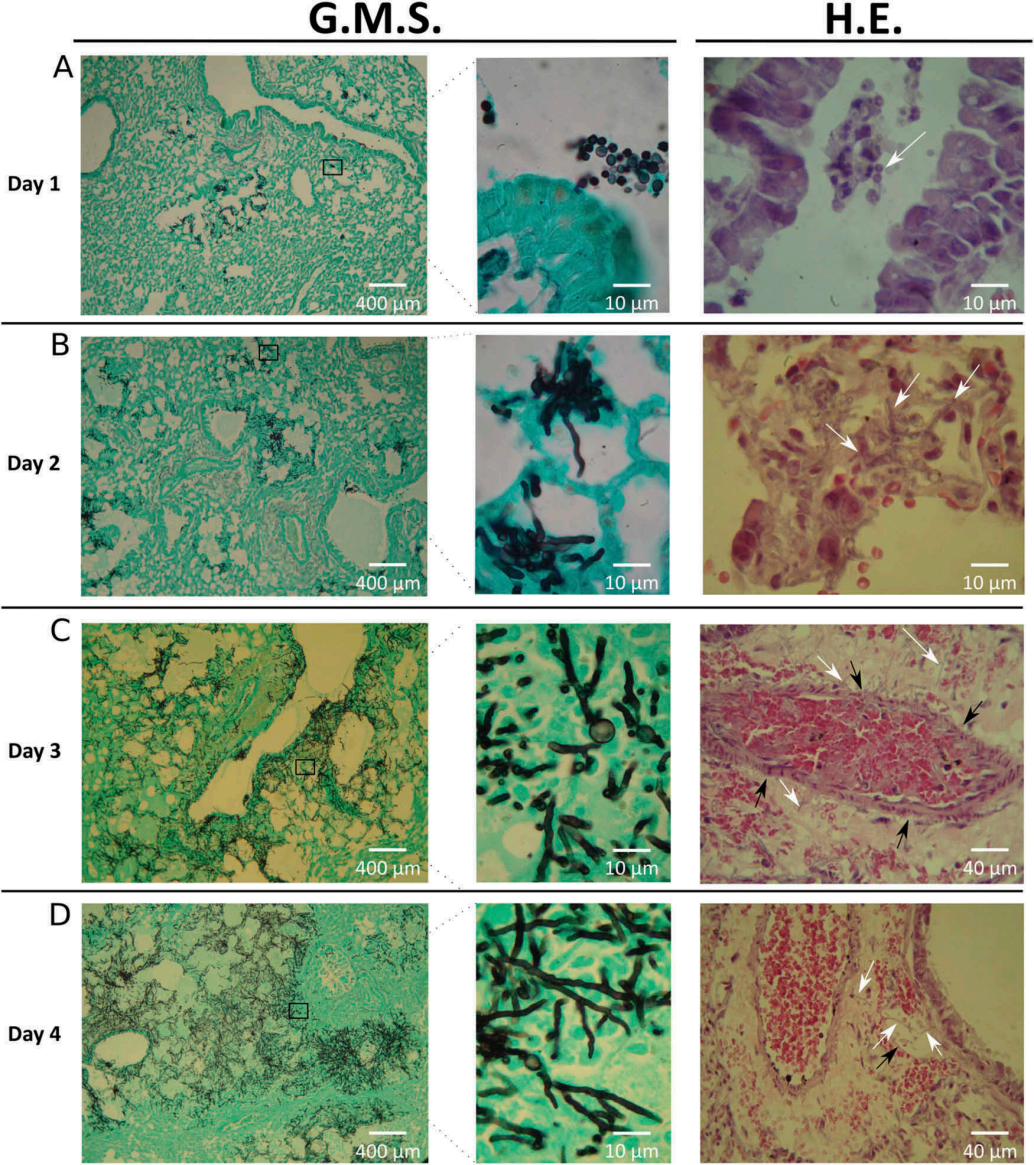
**Histology of infected lungs**. To study the fungal progression and the subsequent tissue damage, lung slices were stained with Grocott’s methenamine silver (G.M.S.) and haematoxylin-eosin (H.E.). White arrows show: (A) conidia in the lumen airways, (B and C) hyphae in lung parenchyma, (D) hyphae in lung parenchyma and inside venous vessels. Black arrows show vascular walls: (C) arterial and (D) venous vessels.

The fungal burden in infected mice was determined by colony forming units (CFU) and histological analysis. Among all organs analyzed, CFUs were only detected in lungs, which showed a progressive decrease in the total number of CFUs recovered over time (data not shown). However, the histological evidence revealed a clear progression of the disease (Figure 2). Specifically, the percentage of lung parenchyma occupied by fungal lesions in each lung sample increased approximately from 5% to 75% during the infection timeline. After the first day, the pathological findings showed most of the conidia around the edge of the airways with small areas of the lung containing germlings (Figure 2A). Over the subsequent days, a rapid progression in the infection process occurred. On the second day post infection, a substantial amount of invasive hyphae were visible, concurrent with the beginning of tissue necrosis (Figure 2B). On the third day, apparent histological necrosis, together with small foci of vascular congestion and hemorrhage, and both arterial and venous angioinvasion obstructing the vascular lumen were seen (Figure 2C). By the fourth day, hyphae had invaded a significant area of lung tissue, encompassing nearly 75% of the parenchyma (Figure 2D). These findings are theoretically compatible with severe respiratory failure and subsequent acute respiratory distress-like syndrome, and death. In no case mice reach this point and always ethical statements were followed. Furthermore, the observed lack of a significant inflammatory response was probably caused by the correct immunosuppression supplied to mice.

### Genes differentially expressed during the progression of lung infection after intranasal exposure to *A. fumigatus* conidia

Of the 9,630 *A. fumigatus* genes represented in the AWAFUGE microarray, only 103 were statistically differentially expressed genes (DEGs) (*p* < 0.05) on day 3 and 4 (72 and 96 h) compared to the first day post infection (24 h).

**Figure 3.**
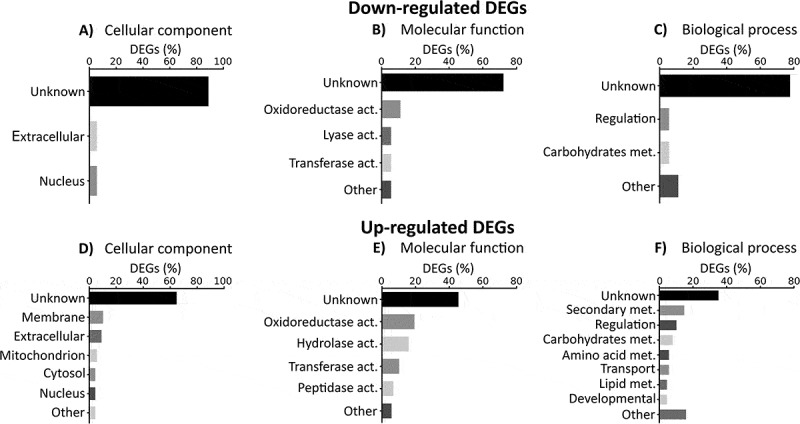
**Gene ontology enrichment of differential expressed genes (DEGs)**. Percentage of down-regulated and up-regulated genes belonging to one of GO categories: (A and D) cellular components, (B and E) molecular functions and (C and F) biological process. The graphics only included the most representative groups (> 4%). Raw data are included in the table S4.

Among these, 18 genes were down-regulated during infection, ten of them detected on both the third and fourth day (Table S1). More than 70% of DEGs were not associated with any GO term (Figure 3A, 3B and 3C) (Table S4). In fact, regarding the concrete function of each down-regulated DEG, 61% (11 out of 18) codify for hypothetical proteins. The rest of the genes encoded two pectin lyases (Afu5g10170 and Afu5g10380), one C6 transcription factor (Afu5g00950), one a dehydrogenase involved in the biosynthesis of the ergot alkaloid fumigaclavine, (*fgaDH* gene, Afu2g18000) [25], one oxidase (Afu3g09500), one acetyltransferase (Afu1g09260) and an ankyrin repeat protein (Afu7g08610) (Table S1) (Table S4).

More numerous than the former group of genes were 85 genes found to be up-regulated (Table S2). Of these, 41 genes were up-regulated at 72 h post-intranasal exposition, 78 genes at 96 h and 33 genes on both days, compared with 24 h. In the GO enrichment study of biological process of this group of genes, more variability in the categories than down-regulated genes were observed in spite of the fact that more than 40% of annotations were also unknown (Figure 3). Around 20% of the up-regulated DEGs were related to fungal structural components such as cytosol, nucleus, cell wall and plasma membrane, while only 5% were related to mitochondria. Interestingly, 20% of the DEGs were predicted to be involved in molecular lytic functions (hydrolase and peptidase activities) and around 15% in secondary metabolism (Figure 3D, 3E and 3F) (Table S4).

Remarkably, the up-regulated DEGs group contained 14 genes (out of 21) of the intertwined fumagillin/pseurotin gene cluster (Table 1). These included the fumagillin/pseurotin transcription factor FapR [24] (also known as FumR [26]), four fumagillin biosynthetic genes, one of the two non-canonical methionine aminopeptidase encoding genes located inside the supercluster region, implicated to convey fumagillin resistance (*fpaII*; Afu8g00410), five pseurotin biosynthetic genes and three genes of unknown function. Additional DEGs included genes codifying for hydrolytic enzymes such as DppIV (Afu4g09320), DppV (Afu2g00930), the allergens Asp f 1 (Afu5g02330) and Asp f 5 or elastinolytic metalloproteinase Mep (Afu8g07080) and the chitinase ChiB1 (Afu8g01410), among others (Table S2).

**Table 1.** Results of Differential Expressed Genes (DEGs) detected using microarray technologies versus expression data using specific primers in RT-qPCR.

			Fold Change^a^			
			Day3 vs Day1		Day4 vs Day1	
Product description^b^	Systematic Name^c^	Standard Name^d^	AWAFUGE	RT-qPCR	AWAFUGE	RT-qPCR
**Fumagillin/Pseurotin pathway**						
Polyketide synthase	Afu8g00370	*fma-PKS / fmaB*		6.28	5.66	7.10
O-methyltransferase	Afu8g00390	*fmaD*	6.37	7.97	5.71	8.46
Hypothetical protein	Afu8g00400		7.8	15.90	6.84	16.23
Methionine aminopeptidase. type II	Afu8g00410	*metAP / fpaII*	4.53	5.03	4.09	4.88
C6 finger transcription factor	Afu8g00420	*fumR / fapR*	4.28	5.48		5.52
Hypothetical protein	Afu8g00430		7.21	9.52	6.97	9.61
Steroid monooxygenase	Afu8g00440	*psoF*	5.22	8.63	4.89	9.05
Phytanoyl-CoA dioxygenase family protein	Afu8g00480	*fmaF*	4.63	11.24	4.65	11.56
Acetate-CoA ligase	Afu8g00500		5.03	9.95	4.68	10.36
Cytochrome P450 oxidoreductase OrdA-like	Afu8g00510	*fmaG*	5.87	11.07	5.02	10.92
α/β hydrolase	Afu8g00530	*psoB*	5.41	8.30	5.29	8.47
Methyltransferase	Afu8g00550	*psoC*	7.09	10.12	6.90	10.70
Cytochrome P450 oxidoreductase	Afu8g00560		4.61	ND	4.46	ND
Glutathione S-transferase like	Afu8g00580	*elfB / psoE*	5.06	9.48	4.96	9.87
**Lytic enzymes**						
Secreted dipeptidyl peptidase DppV	Afu2g09030	*dppV*	3.47	3.25	3.92	3.83
Vacuolar carboxypeptidase Cps1	Afu3g07040	*cps1*			2.82	3.43
Extracellular dipeptidyl-peptidase Dpp4	Afu4g09320	*dppIV*			3.50	0.63
Extracellular lipase	Afu5g02040		3.93	3.20	4.21	3.97
Major allergen and cytotoxin AspF1	Afu5g02330	*aspf1*			4.64	4.09
Lipase	Afu7g04020		5.64	3.62	5.95	4.90
Class V chitinase ChiB1	Afu8g01410	*chiB1*			3.50	5.17
Elastinolytic metalloproteinase Mep	Afu8g07080	*Mep*			3.88	3.59
**Others**						
Acetyltransferase. GNAT family family	Afu1g09260		−3.40	1.43	−3.05	4.12
Hypothetical protein	Afu1g10450		−3.39	−0.41	−3.39	−0.23
Methionine aminopeptidase. type II	Afu2g01750		3.60	2.37	4.10	3.02
4-hydroxyphenylpyruvate dioxygenase	Afu2g04200	*hppD*			4.21	4.87
Maleylacetoacetate isomerase MaiA	Afu2g04240	*maiA*			3.72	3.79
Hypothetical protein	Afu2g16440		−3.35	0.58	−3.66	0.73
Short chain dehydrogenase/ oxidoreductase CpoX2	Afu2g18000	*fgaDH*	−3.43	1.01	−3.31	−0.17
Hypothetical protein	Afu3g00410				−3.47	0.06
MFS sugar transporter	Afu3g03700		3.39	2.31	3.77	3.65
C6 sexual development transcription factor NosA	Afu4g09710	*rosA*			3.87	6.59
C6 transcription factor	Afu5g00950		−3.71	1.26	−4.09	0.72
Hypothetical protein	Afu5g08800		3.99	4.82		
Pectin lyase	Afu5g10170				−3.79	−4.50
Pectin lyase	Afu5g10380				−3.57	−3.31
C6 transcription factor	Afu5g14290		4.36	1.16		
Aldehyde dehydrogenase	Afu7g01000				4.11	7.86
Indoleamine 2.3-dioxygenase	Afu7g02010		4.19	5.94	5.72	8.21
Defensin domain protein	Afu7g05180		5.06	6.71	4.81	4.21
Integral membrane protein Pth11-like	Afu7g06620		3.41	4.72	3.38	4.41

^a^This value represents the difference of the fold change in log_2_ obtained for each gene between days of infection compared in each case. A negative or positive value indicated down or up-regulation relative to the first day post-infection. respectively. AWAFUGE: data obtained with Agilent Whole *A. fumigatus* Genome Expression 44K v.1; RT-qPCR: data obtained with RT-qPCR using the *A. fumigatus* specific primers designed.

^b^Product description of the genes found on the microarray following RefSeq nomenclature.

^c^Systematic name of the gene following AspGD nomenclature.

^d^Gene name following AspGD nomenclature (http://www.aspergillusgenome.org). except fumagillin/pseurotin pathway that also follow the nomenclature published by Wieman P. *et al*. (2013).

### RT-qPCR verification of microarray data

Since traces of mouse RNA in analyzed microarray samples could remain and lead to bias due to cross-hybridization phenomena, a RT-qPCR confirmation assay was designed to estimate the accuracy of the obtained microarray results.

**Figure 4.**
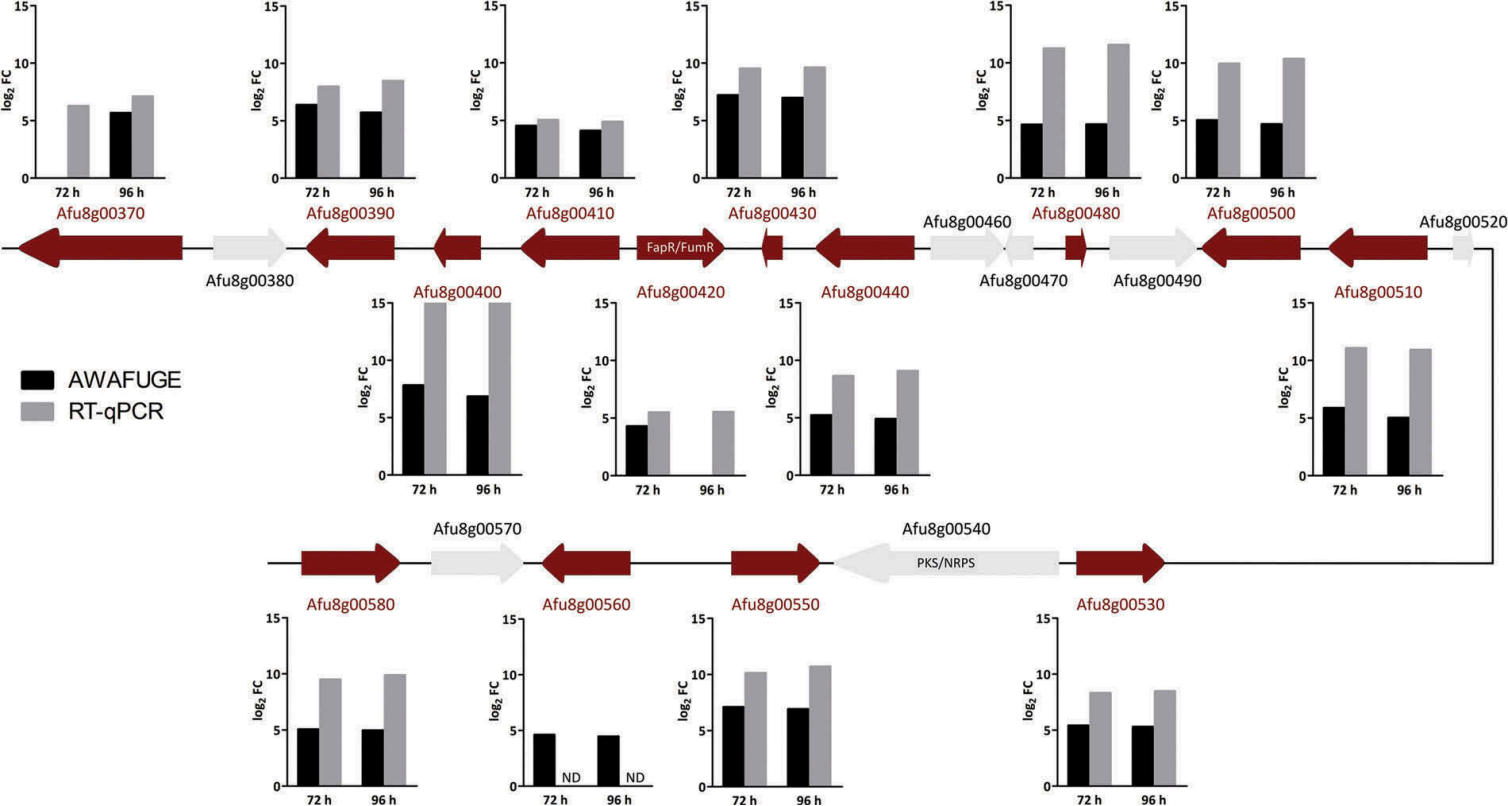
**Schematic representation of the intertwined fumagillin/pseurotin gene cluster**. Red arrows indicate the up-regulated DEGs relative to the first day post-infection. Graphics represent the log_2_ fold change (FC) obtained by AWAFUGE microarray and RT-qPCR assays. FumR/FapR is the transcription factor that regulates fumagillin and pseurotin production. PKS/NRPS regulates pseurotin production. ND: Not determined by RT-qPCR.

Forty-eight genes were confirmed by RT-qPCR, including most of those related to the production of fumagillin and pseurotin (Figure 4), the hydrolytic enzymes, a few transcription factors and some metabolic enzymes detected on the microarray. The analysis revealed that 92% of the genes selected exhibited the same expression pattern as the microarray (Table 1). In addition, the genes selected as fungal housekeeping (Afu2g02920, Afu3g013950, Afu3g14500 and Afu7g01580) did not vary in their expression, and none of the *Aspergillus* specific primers showed RT-qPCR amplifications in the non-infected mouse RNA samples (data not shown).

### Fumagillin production is associated with cytotoxicity in an *in vitro* infection model of a pneumocyte cell line

The observation that over half of the fumagillin/pseurotin cluster genes were significantly upregulated during infection led us to query if either metabolite could be elucidating/causing a host response. To address a possible role of either metabolite in host cell damage, we analyzed the interactions with the A549 pulmonary epithelial cell line of wild type *A. fumigatus*, a fumagillin mutant strain, *∆fmaA*, which still produces pseurotin, and a pseurotin mutant strain, *∆psoA*, which still produces fumagillin [24].

**Figure 5.**
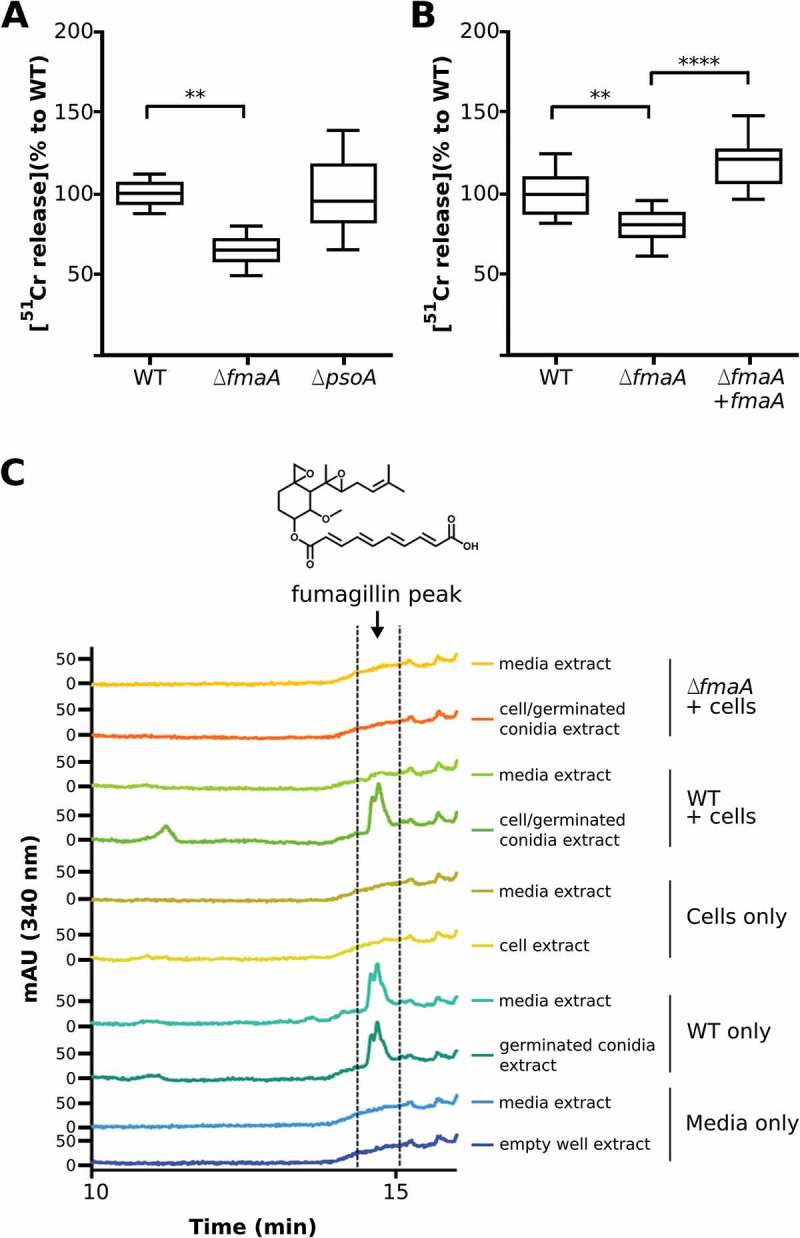
**Cell damage and fumagillin production during co-incubation of *A. fumigatus* with A549 cells**. (A and B) Extent of *A. fumigatus-*induced damage to the A549 pulmonary epithelial cell line. The indicated strains of *A. fumigatus* were incubated with A549 cells for 20h, and the extent of epithelial cell damage was determined using a ^51^Cr release assay. Results in (A) are a box-whisker plot of three independent experiments, each performed in duplicate. Results in (B) are from three independent experiments, each performed in triplicate. **p < 0.01; **** *p *< 0.0001. (C) HPLC chromatograms of indicated sample fractions from A549 cells co-incubated with *A. fumigatus* conidia after 24 h and respective controls (cells without *A. fumigatus* conidia, conidia without cells and media only). Samples were prepared as described in Material and Methods. The wavelength of the shown chromatograms was set to 340 nm, which is the absorption maximum for fumagillin detected at 14.5 min. Fumagillin was absent in the ∆*fmaA* mutant.

First, the extent of epithelial cell injury was assessed by the ^51^Cr release method and the cytotoxic effect of each mutant was compared with the WT strain (Figure 5A). After 20 h of infection, the *∆fmaA* strain caused significantly less epithelial cell injury than the wild-type strain, while the damage caused by *∆psoA* strain was similar to that of the WT. In a separate set of experiments, complementing the *∆fmaA* strain with an intact copy of *fmaA* was found to restore its capacity to damage the epithelial cells (Figure 5B).

Next, we determined whether fumagillin was detected in association with A549 cells during co-incubation with *A. fumigatus*. For the WT strain, fumagillin could be detected in the absence and in presence of epithelial cells, while no fumagillin was detected from samples using the ∆*fmaA* strain or the cell and media controls only, respectively (Figure 5C). Interestingly, in presence of epithelial cells, fumagillin was only detectable in the cell lysate but not in the media (Figure 5C) while, in the absence of cells, fumagillin was detected in media when the *A. fumigatus* WT was grown. These data suggest a localization of fumagillin to A549 cells during co-incubation with *A. fumigatus*.

## Discussion

Fair interpretation of host-pathogen interactions and invasive aspergillosis pathogenesis requires a rigorous understanding of gene expression changes that occur during the disease progression with follow up on protein or metabolite production. Therefore, in this work, we focused on these alterations during invasive lung aspergillosis after an intranasal exposure to *A. fumigatus* conidia using a murine model with a focus on gene expression and metabolite bioactivity of the fumagillin/pseurotin gene cluster. In this way, this study might contribute to the understanding of the primary mechanisms used by *A. fumigatus* to develop an infection once conidia have entered and settled in the murine lung.

During the lung infection process, our pathological study revealed that pulmonary aspergillosis progressed daily. At the beginning of the infection, *Aspergillus* conidia were detected inside the airways, distributed into small infection loci filled with fungal germlings eliciting a low or no inflammatory response. After 24 h, vigorous growth and spread of the fungal cells occurred, including hyphae formation, causing severe respiratory tissue disruption and blood vessel angioinvasion. This progression is consistent with observations made by other studies [27]. Concurrent with the invasion process, genes that showed differential expression between the moment when the fungus invades tissue and penetrates blood vessels (72 and 96 h post infection) compared to the beginning of the infection (24 h) were analyzed.

Of the whole genome, only 103 genes – approximately 1% of total *A. fumigatus* genes – were detected as differentially expressed. Other studies reported that transcriptional changes during the initiation of invasive aspergillosis involve more than 1,000 genes. However, in those studies, the authors compared fungal growth *in vivo* (during infection) *vs. in vitro* (on culture media) [21,22]. Therefore, the interpretation of these results and the subsequent conclusions have to be taken with caution, since transcriptomic studies depend on the experimental design, the time points studied and the type of reference sample used [28]. Our study was designed so that the first day of infection was used as reference, this was to avoid comparisons with *in vitro* conditions which may have led to differences connected to the culture conditions but not to the infectious process. In this way, we try to explain the transcriptomic changes during the progression of the infection, and we ignore the adaptive process (first 24 h).

To avoid biased interpretations due to cross-hybridization with traces of mouse RNA that could remain in some samples, we confirmed the result of the most relevant genes identified after microarray analysis by RT-qPCR using fungal-specific primers. Our results support the utility of the microarray data as an initial screening tool to point out the most relevant genes during infection progression. In fact, 92% of the genes identified by microarray analysis could be confirmed by specific RT-qPCR. This assessment is particularly critical because most of the detected genes in our study have been implicated in lung invasive aspergillosis previously [20,29,30].

As observed in other studies, we found that many of the differentially regulated genes encoded for hypothetical proteins (61% of the down-regulated genes – 11 genes – and 22% – 19 genes – of the up-regulated genes). Thus, these proteins may play an important role during the initial stages of lung infection as well as during the progression of the lung infection.

Of the few down-regulated DEGs with putative functions, two encoded pectin lyases, which are essential for the degradation of pectins during plant infection. Other authors describe *A. fumigatus* as a microorganism better adapted to plant decomposition than to human infection [31], so these genes might show a high basal expression, which drop dramatically during a human infection.

Most of the DEGs showed significant increase in expression during the progression of the infectious disease. Among them, the 14 (out of 21) genes belonging to the intertwined fumagillin/pseurotin biosynthetic cluster were notable, including the essential transcription factor FumR/FapR (Afu8g00420) critical for the production of both metabolites [24,26]. Our transcriptome data showed that *fapR* expression was early with increases of expression of the *fma/pso* biosynthetic genes with invasion time. Although several *A. fumigatus* secondary metabolites such as gliotoxin [32] and endocrocin [33] have been shown to contribute to *A. fumigatus* virulence or interactions with immune cells, no studies have addressed any effects of either fumagillin or pseurotin in this regard. However the anti-angiogenic effects of fumagillin are well known [34,35], reducing the proliferation of endothelial cells and blood vessel formation [36–40]. Angiogenesis has been proposed as a host response to thrombosis and necrosis caused by *A. fumigatus* angioinvasion, in order to compensate for hypoxia and to achieve higher levels of effector molecules at the infection site [41]. Additionally, this mycotoxin promotes epithelial cell damage and slower beating of ciliary cells [42–45]. Purified fumagillin was shown to inhibit the immune response of larvae of the wax moth *Galleria mellonella* [46]. In contrast, pseurotin, which has been poorly studied, had anti-inflammatory properties [47].

To examine any possible effect of fumagillin production by *A. fumigatus* on host cells, we compared the extent of damage to a pulmonary epithelial cell line following incubation with either the wild-type strain, a known fumagillin mutant, *∆fmaA*, or a known pseurotin mutant, *∆psoA*, of *A. fumigatus*. Importantly, we found that the fungus does produce fumagillin in cell cultures and that the *∆fmaA* mutant caused less epithelial cell damage than the wild-type strain. Our results also showed that this toxin is undetectable in the supernatant of infected cultures, indicating that it is retained on cell surface or inside the cells, thus being able to exert its toxic function. In addition, fumagillin added to A549 pulmonary epithelial cells culture disappeared progressively from the media, decreasing the concentration up to 80% in 24 h compared with controls without cells (data not sown). Moreover, it is highlighting that fumagillin was detected by HPLC in *in vitro* assays at 24 h, while transcriptomic data of *in vivo* infections showed up-regulation at 72 h compared with 24 h. Therefore, although fumagillin seems to be produced during the early stages of infection, its expression increases as the infection progresses. However, animal infections with the abovementioned mutant and complemented strains will be necessary in a near future to deepen into the knowledge about this mycotoxin.

Other up-regulated DEGs encoded putative lytic enzymes, which may be related to the invasion and subsequent destruction of the lung parenchyma. It is well known that *A. fumigatus* produces a wide range of proteases and hydrolytic enzymes to degrade the lung and liberate nutrients to enable fungal growth [21,31,48]. However, more information is required to understand which proteases and enzymes have a role in providing nutrients to *A. fumigatus* in the human lungs [31]. Regarding this group of genes in our study, two important lipase encoding genes were identified (Afu5g02040 and Afu7g04020). Lipases seems to be important molecules in the host-pathogen interaction processes [49]. Additionally, four of the major extracellular proteases secreted by *A. fumigatus* (DppIV, DppV, Mep or Asp f 5, and the ribotoxin Asp f 1) were also found overexpressed at the onset of our infection model. Some of them, are well-known allergens and virulence factors. In fact, the expression of Asp f 1 is greater when the transcriptomic data obtained from mice infection models are compared to *in vitro* assays [50,51]. In addition to these findings, the detection of another gene that encodes a major chitinase (chiB1), which is related to autolytic functions [52,53], could indicate a partial cell wall lysis allowing the hyphal elongation during progression of invasion. Finally, it is remarkable that many of the proteins codified by these genes, such as DppV, chitinase, Asp f 5 or Asp f 1, have been previously reported as secreted antigens related to infection and are of interest for medical applications [54].

Furthermore, two of the six genes of the tyrosine degradation cluster (*hppD* and *maiA*) and an aldehyde dehydrogenase (Afu7g01000), a gene that is overexpressed in the presence of L-tyrosine [55], were DEGs, with a higher expression on the final day of infection. Interestingly, pyomelanin is derived by degradation of L-tyrosine present in the lungs of immunosuppressed patients, and has been reported to protect swollen conidia and young hyphae from reactive oxygen intermediates [56]. Therefore, our results suggest that *A. fumigatus* could synthesize pyomelanin during lung infection to protect fungal cells during progression of invasion, and ensure an efficient dispersion. However, as only two genes of pyomelanin biosynthesis were detected in our study, further experiments should be carried out to confirm this hypothesis.

**Figure 6.**
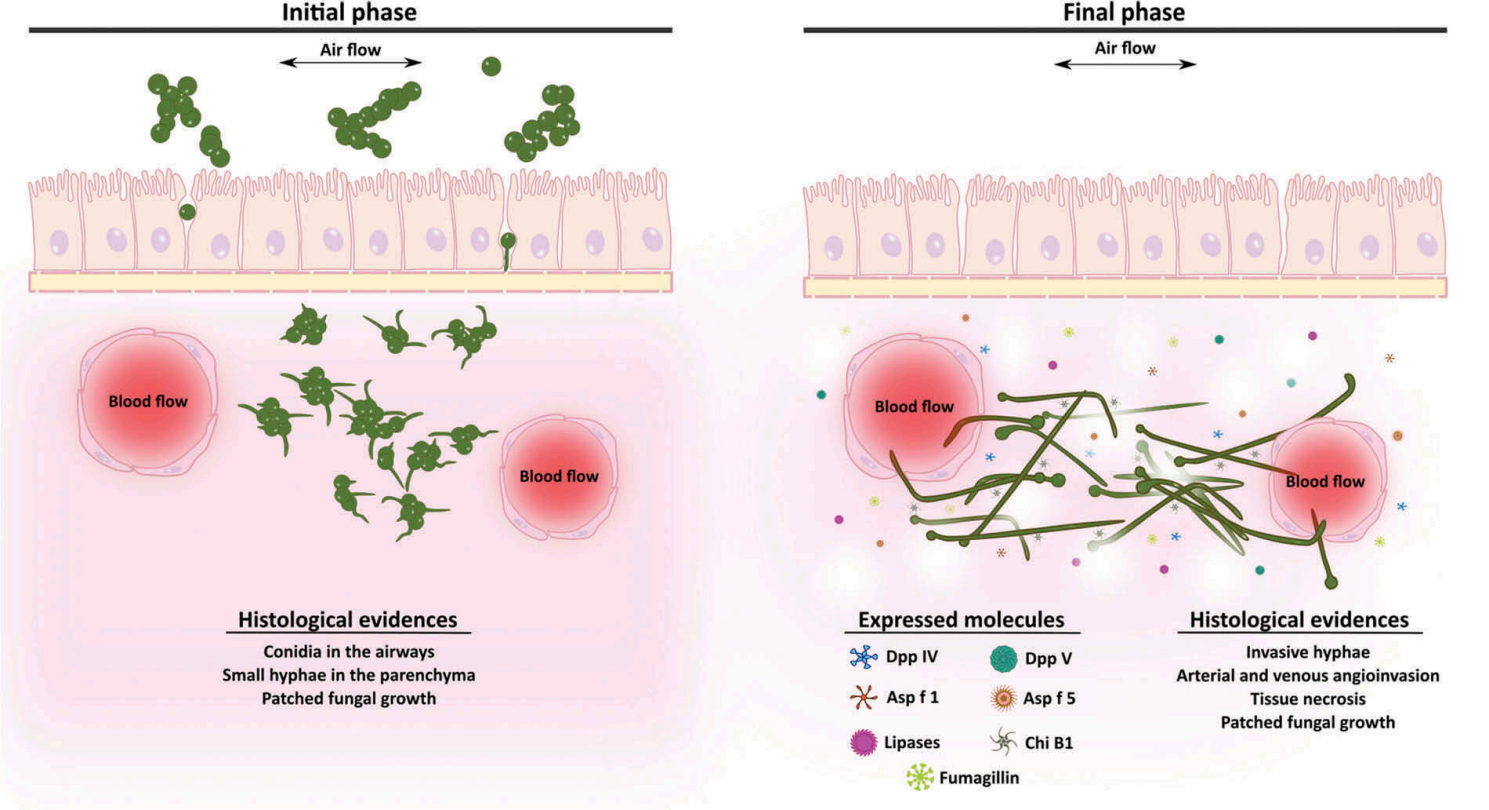
**Scheme of the development of an *Aspergillus fumigatus* intranasal infection in a murine model**. In the initial stages of the infection (1st and 2nd days), fungal conidia colonize the lung tissue and start to germinate invading the parenchyma. By contrast, during the final phase of the infection (3rd and 4th days), which are associated with angioinvasion and tissue necrosis, the fungus continues growing and forming invasive hyphae and increasing the expression of genes related to virulence such as lytic enzymes and fumagillin.

In conclusion, this study suggests that gene expression and pathological changes are correlated during the development of invasive lung aspergillosis. The up-regulation of genes encoding lytic enzymes and fumagillin biosynthetic enzymes could promote angioinvasion, epithelial destruction, and lung parenchymal architecture disruption (Figure 6). To our knowledge, this is the first study to propose fumagillin may be an important contributor during early stages of infection. This strategy may allow the fungus to evade some host defense mechanisms and promote fungal invasion in order to reach other organs and tissues.

## Methods

### *Aspergillus fumigatus* strains, media and growth conditions

The Af293 strain of *A. fumigatus* was grown on potato dextrose agar (Cultimed, Castellar de Vallés, Spain) at 28°C for seven days to obtain conidia for infection. After harvesting and cleaning twice with a saline-Tween solution (0.9% NaCl, 0.02% Tween 20) (SS-Tw20), the number of conidia was calculated using a haemocytometer and their viability tested by plating them onto potato dextrose agar (Cultimed) and expressed as a percentage. Furthermore, the previously published [24] deletion mutants strains *∆fmaA* (fumagillin^−^ pseurotin^+^) and *∆psoA* (fumagillin^+^ pseurotin^−^) were used for the fumagillin ^51^Cr release assays and HPLC detection assays, alongside their correspondent wild type strain *A. fumigatus* CEA17.

### Complementation of *∆fmaA*

The extraction of DNAs from fungi and bacteria, restriction enzyme digestion, gel electrophoresis, blotting, hybridization, and probe preparation were performed by standard methods [57,58]. pJW165 for *ΔfmaA* complementation was generated by standard techniques. Pfu Ultra II Fusion HS DNA polymerase (Agilent, Santa Clara, USA) was used to amplify a 3 kb fragment by fmacomhygF and fmacomhygR (Table S3), which includes *fmaA* wild type gene with its own promoter and terminator from *A. fumigatus* CEA10. This 3kb amplicon was also used as a template for Southern hybridization probe. The PCR product was inserted into the *Hind*III site of pUG2-8 [59], which contains a hygromycin B (*hygB*) resistance gene. The *fmaA* gene in pJW165 was confirmed by sequencing before transformation as the previously published transformation method [57]. Transformants were selected in hygromycin B (100 μg/ml)-containing medium and confirmed by Southern hybridization (Supplementary Figure 1). We also confirmed the restoration of fumagillin in the complemented strain TJW199.14 (data not shown).

### Animal infection

First of all, an assay to select the suitable *A. fumigatus* dose was performed. For that, 1 x 10^6^, 1 × 10^7^ and 1 × 10^8^ *A. fumigatus* resting conidia per mouse were intranasally administered and survival rates studied.

Once the suitable dose was selected, three independent animal infections were carried out. For each one, ten female BALB/c mice between 16–20 g were used. Mice were kept in the General Animal Unit Service of the University of the Basque Country (UPV/EHU), with water and food *ad libitum*, handled in biological safety cabinets, and kept in sterilized cages with negative-pressure ventilation and filters. All the mice were immunosuppressed by the administration of 150 mg/Kg cyclophosphamide (Sigma-Aldrich, Madrid, Spain) intraperitoneally four days and 100 mg/Kg the day before infection. Eight mice were infected by exposing them intranasally to a 20 µl SS-Tw20 solution containing 1 × 10^7^ resting conidia of *A. fumigatus*. The other two animals received 20 µl of SS-Tw20 without conidia and were used as controls. After infection, two animals were euthanized daily, minimizing mice suffering. Controls were euthanized on the fifth day. Subsequently, the pair of lungs of each mouse were extracted and divided into two halves. One half was used for histological diagnosis and colony forming units (CFUs) counting onto Sabouraud glucose agar with chloramphenicol plates (Cultimed). The other half was pooled with the half of the lungs of the other mouse euthanized the same day to isolate total RNA, which was kept at −80ºC into RNAlater (Qiagen, Valencia, CA and the USA).

### Histological study

Lungs removed from mice were fixed with 10% formalin, and subsequently embedded in paraffin. After this, consecutive slices four micrometers in thickness were obtained and stained with haematoxylin-eosin (H.E.) and Grocott´s silver methenamine (G.M.S.) to carry out a classic fungus histology study.

### RNA isolation

Mice lung tissues conserved in RNAlater were broken and homogenized by blunt crushing in a bag (Deltalab, Barcelona, Spain), that contained 2 ml of DEPC sterile water. The resulting material was centrifuged at 13,000 g for 3 minutes, and the pellet obtained was ground three times in the presence of liquid nitrogen using an agate mortar. Finally, total RNA was isolated using the RNeasy Plant Mini Kit (Qiagen) following the manufacturer’s protocol, and the RNA quantity and integrity verified on a 2100 Bioanalyzer (Agilent Technologies, Santa Clara, CA, USA). For transcriptomic studies, microarray analysis and RT-qPCR confirmation, three independent RNA samples for each time point, each of them obtained from an independent infection, were studied.

### Microarray selection and hybridization

The Agilent Whole *A. fumigatus* Genome Expression 44K v.1 (AWAFUGE) microarray was used to analyze the transcriptome profiles from each sample [17]. From each time point studied, starting at a maximum of 100 ng per sample, RNA was labeled using the “Low Input Quick Amp WT Labeling kit, One-Color” kit (Agilent Technologies, Santa Clara, CA, USA). Then, cDNA was transcribed using T7 RNA polymerase in the presence of Cy3-CTP, and hybridized using the SureHyb hybridization chambers (Agilent Technologies) following the ozone barrier slide covers Agilent protocol. Finally, microarray slides were scanned using a GenePix 4100A scanner (Axon Instruments), and images were analyzed using the associated GenePix Pro 6.0 software (Molecular Devices).

### Microarray expression data analysis

The raw intensity data obtained from each microarray was processed following the conventional scheme, using the limma library under the Bioconductor package [60,61]. After subtracting the background and normalizing the data set using the normexp and quantile routines, respectively, expression levels were compared using an ANOVA test with a Benjamini-Hochberg correction. The statistical significance level was fixed at 0.05. Genes were considered down-regulated or up-regulated if their expression was significantly lower or higher relative to the first day. Results were expressed as log_2_ fold change, in the case of the DEGs the log_2_ fold change represents the statistical difference of expression between the first day of infection and the third and fourth days. A negative or positive value indicated down or up-regulation relative to the first day post-infection, respectively.

### Gene ontology (GO) analysis

The GO enrichment of differentially expressed genes was performed using the GO Slim Mapper tool available in the *Aspergillus* Genome Database (available at http://www.aspergillusgenome.org). Most of them were ascribed to one of the three GO domains (Cellular Component, Molecular Function, and Biological Process), regardless of their expression pattern.

### Microarray data confirmation by reverse transcription quantitative PCR

The genes selected were verified by RT-qPCR. To avoid false positives due to mouse RNA remaining in the samples, *A. fumigatus* specific primers were designed using Primer Quest Tool (available at https://eu.idtdna.com/site) (Table S3). RT-qPCR experiments and subsequent data analysis were performed following the methodology described by Sueiro-Olivares et al. [17].

### ^51^Cr release cytotoxic assay

The amount of epithelial cell injury induced by conidia and hyphae was quantified by the release of ^51^Cr. Briefly, A549 pulmonary epithelial cell line were grown to confluence in 96-well plates containing detachable wells. The cells were incubated overnight with 1 µCi (0.037 MBq) Na_2_^51^CrO4 (ICN Biomedicals, Irvine, CA) per well. The following day, the unincorporated tracer was aspirated and the wells were rinsed twice with prewarmed Hanks balanced salt solution. Next, epithelial cells were incubated with 10^5^ spores per well in 100 µL tissue culture medium using either wild type *A. fumigatus* or the various mutant strains.

After 20 h on incubation, the upper 50% of the medium was aspirated from each well and then the wells were manually detached from one another. The amount of ^51^Cr in the aspirates and in the wells was determined by gamma counting. To measure the spontaneous release of ^51^Cr, uninfected epithelial cells exposed to medium alone were processed in parallel as control. For the initial screen of the mutants, each strain was tested in duplicate in 3 separate experiments. When the Δ*fmaA* and Δ*fmaA*+*fmaA* complemented strains were tested, each strain was tested in triplicate in 3 independent experiments. The percentage of specific release of ^51^Cr was calculated as previously described [62].

### Fumagillin detection

For fumagillin analysis, co-incubation of cells and fungal strains were carried out as described in the cell damage assay above and samples from the 24 h time points were processed as follows. A549 pulmonary epithelial cells were incubated with 10^5^ conidia of either the WT or the ∆*fmaA* per well in 100 µL tissue culture medium for 24 h. As controls, cells without conidia, conidia without cells, and only media were used under the same conditions. Cells/germinated conidia and media were separated by centrifugation. Cells/germinated conidia were washed twice and resuspended in 500 µL media. Both, cells and supernatant were extracted with an equal amount of ethyl acetate, respectively. Ethyl acetate was evaporated and samples were resuspended in methanol and filtered through 0.2 µm polyvinylidene fluoride filters before high performance liquid chromatography (HPLC) photodiode array (PDA) analysis. The samples were separated on a ZORBAX Eclipse XDB-C18 column (Agilent, 4.6 mm by 150 mm with a 5 μm particle size) using a binary gradient of 0.5 % (v/v) formic acid (FA) as solvent A and 0.5 % (v/v) FA in acetonitrile (ACN) as solvent B delivered by a Flexar Binary Liquid Chromatography (LC) Pump (PerkinElmer) coupled to a Flexar LC Autosampler (Perkin Elmer) and a Flexar PDA Plus Detector (PerkinElmer). The PDA was set to 340 nm which is the absorption maximum for fumagillin. Identification of fumagillin was performed using Chromera Manager (PerkinElmer) by comparison to UV peak patterns and retention time of standards, as described by Wiemann et al. [63]. The fumagillin showed a peak detected at 14.5 min.

### Availability of data

The ArrayExpress database contains the AWAFUGE microarray (v.1) design under accession number A-MEXP-2352. The same database also contains each raw microarray dataset obtained under accession number E-MTAB-5314.

### Ethical issues

The Ethics Committee for Animal Welfare (CEBA) of the University of the Basque Country (UPV/EHU) (reference number CEBA/36-P03-01/2010/REMENTERIA RUIZ and CEBA/36-P03-03/2010/REMENTERIA RUIZ) approved all the animal experimental procedures carried out in this study.
