# Supplementary material for: A possible role for fumagillin in cellular damage during host infection by Aspergillus fumigatus
Source: Virulence. 2018 Sep 25;9(1):1548–61. doi: 10.1080/21505594.2018.1526528 (PMC6177242; doi:10.1080/21505594.2018.1526528)
Supplement: Supplemental Material [file kvir-09-01-1526528-s001.zip › Table S1_down-regulated_DEGs.docx]

**Table S1.** Results of 18 DEGs down-regulated detected after microarrays analysis

|  |  |  | **Fold Change^a^** | |
| --- | --- | --- | --- | --- |
| **Product description^b^** | **Systematic Name^c^** | **Standard Name^d^** | **Day3 vs Day1** | **Day4 vs Day1** |
| Hypothetical protein | Afu3g14390 |  | -4.72 | -5.17 |
| Hypothetical protein | Afu8g06300 |  | -3.98 | -2.94 |
| C6 transcription factor | Afu5g00950 |  | -3.71 | -4.09 |
| Hypothetical protein | Afu7g07140 |  | -3.57 | -4.08 |
| Hypothetical protein | Afu1g12475 |  | -3.53 | -3.67 |
| Short chain dehydrogenase/ oxidoreductase CpoX2 | Afu2g18000 | *fgaDH* | -3.43 | -3.31 |
| Acetyltransferase. gNAT family family | Afu1g09260 |  | -3.4 | -3.05 |
| Hypothetical protein | Afu1g10450 |  | -3.39 | -3.39 |
| Hypothetical protein | Afu8g01795 |  | -3.39 | -3.21 |
| Hypothetical protein | Afu2g16440 |  | -3.35 | -3.66 |
| Aryl-alcohol oxidase vanillyl-alcohol oxidase | Afu3g09500 |  | -4.64 |  |
| Hypothetical protein | Afu4g08310 |  | -3.73 |  |
| Hypothetical protein | Afu5g14850 |  | -3.49 |  |
| Hypothetical protein | Afu6g03330 |  | -3.41 |  |
| Ankyrin repeat protein | Afu7g08610 |  | -3.37 |  |
| Pectin lyase | Afu5g10170 |  |  | -3.79 |
| Pectin lyase | Afu5g10380 |  |  | -3.57 |
| Hypothetical protein | Afu3g00410 |  |  | -3.47 |

^a^This value represents the difference of the fold change in log_2_ obtained for each gene between days of infection compared in each case. Negative values indicated down-regulation relative to the first day post-infection. Data obtained with Agilent Whole *A. fumigatus* genome Expression 44K v.1.

^b^Product description of the genes found on the microarray following RefSeq nomenclature.

^c^Systematic name of the gene following AspgD nomenclature.

^d^Gene name following AspgD nomenclature (http://www.aspergillusgenome.org).
