# Supplementary material for: A possible role for fumagillin in cellular damage during host infection by Aspergillus fumigatus
Source: Virulence. 2018 Sep 25;9(1):1548–61. doi: 10.1080/21505594.2018.1526528 (PMC6177242; doi:10.1080/21505594.2018.1526528)
Supplement: Supplemental Material [file kvir-09-01-1526528-s001.zip › Table S2_up-regulated_DEGs.docx]

**Table S2.** Results of the 85 DEGs up-regulated detected after microarrays analysis.

|  |  |  | **Fold Change^a^** | |
| --- | --- | --- | --- | --- |
| **Product description^b^** | **Systematic Name^c^** | **Standard Named^d^** | **Day3 vs Day1** | **Day4 vs Day1** |
| Indoleamine 2.3-dioxygenase family protein | Afu7g02010 |  | 4.19 | 5.72 |
| Hypothetical protein | Afu8g00400 |  | 7.80 | 6.84 |
| Hypothetical protein | Afu8g00430 |  | 7.21 | 6.97 |
| Methyltransferase SirN-like | Afu8g00550 | *psoC* | 7.09 | 6.90 |
| O-methyltransferase | Afu8g00390 | *fmaD* | 6.37 | 5.71 |
| Cytochrome P450 oxidoreductase OrdA-like | Afu8g00510 | *fmaG* | 5.87 | 5.02 |
| Lipase | Afu7g04020 |  | 5.64 | 5.95 |
| α/β hydrolase | Afu8g00530 | *psoB* | 5.41 | 5.29 |
| Steroid monooxygenase | Afu8g00440 | *psoF* | 5.22 | 4.89 |
| Glutathione S-transferase | Afu8g00580 | *elfB / psoE* | 5.06 | 4.96 |
| Defensin domain protein | Afu7g05180 |  | 5.06 | 4.81 |
| Acetate-CoA ligase | Afu8g00500 |  | 5.03 | 4.68 |
| Hypothetical protein | Afu6g01870 |  | 4.97 | 4.65 |
| Phytanoyl-CoA dioxygenase family protein | Afu8g00480 | *fmaF* | 4.63 | 4.65 |
| Cytochrome P450 oxidoreductase | Afu8g00560 |  | 4.61 | 4.46 |
| Methionine aminopeptidase. type II | Afu8g00410 | *metAP / fpaII* | 4.53 | 4.09 |
| GTP-binding protein EsdC | Afu7g01930 | *esdC* | 4.26 | 3.78 |
| IgE-binding protein | Afu6g00430 |  | 4.18 | 4.02 |
| DUF124 domain protein | Afu3g08610 |  | 4.18 | 3.34 |
| ThiJ/PfpI family protein | Afu5g02670 |  | 4.13 | 4.02 |
| Secreted antimicrobial peptide | Afu8g00710 |  | 4.04 | 4.28 |
| Plasma membrane protein Pth11-like | Afu7g06130 |  | 4.02 | 3.71 |
| β-D-glucoside glucohydrolase | Afu7g06140 | *exg13* | 4.01 | 4.10 |
| Hypothetical protein | Afu6g13740 |  | 3.96 | 3.12 |
| Extracellular lipase | Afu5g02040 |  | 3.93 | 4.21 |
| Hypothetical protein | Afu5g00700 |  | 3.92 | 4.32 |
| Hypothetical protein | Afu2g08820 |  | 3.75 | 3.60 |
| Hypothetical protein | Afu8g00910 |  | 3.68 | 3.45 |
| Methionine aminopeptidase. type II | Afu2g01750 |  | 3.60 | 4.10 |
| Hypothetical protein | Afu3g13080 |  | 3.48 | 4.45 |
| Secreted dipeptidyl peptidase DppV | Afu2g09030 | *dppV* | 3.47 | 3.92 |
| Hypothetical protein | Afu1g09030 |  | 3.45 | 4.00 |
| Integral membrane protein Pth11-like | Afu7g06620 |  | 3.41 | 3.38 |
| MFS sugar transporter | Afu3g03700 |  | 3.39 | 3.77 |
| Cytochrome P450 monooxygenase | Afu6g02210 |  |  | 4.28 |
| Aldehyde dehydrogenase | Afu7g01000 |  |  | 4.11 |
| H+/nucleoside cotransporter | Afu6g13190 |  |  | 4.10 |
| Citrate synthase Cit1 | Afu6g03590 | *mcsA* |  | 4.05 |
| Fumarate reductase Osm1 | Afu8g05530 | *osm1* |  | 3.97 |
| Glycerol kinase | Afu6g08470 |  |  | 3.89 |
| Elastinolytic metalloproteinase Mep | Afu8g07080 | *mep* |  | 3.88 |
| 3-methylcrotonyl-CoA carboxylase beta subunit (MccB). putative | Afu5g08940 |  |  | 3.20 |
| C6 transcription factor | Afu5g14290 |  | 4.36 |  |
| C6 finger transcription factor | Afu8g00420 | *fumR / fapR* | 4.28 |  |
| Hypothetical protein | Afu5g08800 |  | 3.99 |  |
| MFS myo-inositol transporter | Afu4g01560 |  | 3.95 |  |
| Hypothetical protein | Afu4g12700 |  | 3.64 |  |
| Hypothetical protein | Afu5g00590 |  | 3.35 |  |
| LaeA-like methyltransferase | Afu2g04380 |  | 3.00 |  |
| Polyketide synthase | Afu8g00370 | *fma-PKS* |  | 5.66 |
| Major allergen and cytotoxin AspF1 | Afu5g02330 | *aspf1* |  | 4.64 |
| N.O-diacetyl muramidase | Afu6g10130 |  |  | 4.4 |
| Stress responsive A/B barrel domain protein | Afu4g10610 |  |  | 4.39 |
| 4-hydroxyphenylpyruvate dioxygenase | Afu2g04200 | *hppD* |  | 4.21 |
| Hypothetical protein | Afu1g02290 |  |  | 3.91 |
| C6 sexual development transcription factor NosA | Afu4g09710 | *rosA* |  | 3.87 |
| NAD-dependent formate dehydrogenase AciA/Fdh | Afu6g04920 | *fdh* |  | 3.82 |
| Hypothetical protein | Afu2g17630 |  |  | 3.75 |
| Maleylacetoacetate isomerase MaiA | Afu2g04240 | *maiA* |  | 3.72 |
| Succinyl-CoA:3-ketoacid-coenzyme A transferase (ScoT). putative | Afu6g12250 |  |  | 3.61 |
| DUF614 domain protein | Afu1g14190 |  |  | 3.58 |
| NAD+ dependent glutamate dehydrogenase | Afu2g06000 |  |  | 3.53 |
| Class V chitinase ChiB1 | Afu8g01410 | *chiB1* |  | 3.50 |
| Extracellular dipeptidyl-peptidase Dpp4 | Afu4g09320 | *dppIV* |  | 3.50 |
| Fg-GAP repeat protein | Afu1g04130 |  |  | 3.49 |
| Hypothetical protein | Afu5g10930 |  |  | 3.48 |
| Carbon-nitrogen family hydrolase | Afu4g02790 |  |  | 3.47 |
| WSC domain protein | Afu3g07050 | *wsc2* |  | 3.45 |
| DUF895 domain membrane protein | Afu1g00440 |  |  | 3.39 |
| Homoserine acetyltransferase family protein | Afu1g15350 |  |  | 3.38 |
| Hypothetical protein | Afu1g15160 |  |  | 3.38 |
| Hypothetical protein | Afu5g02320 |  |  | 3.33 |
| MFS transporter | Afu2g17840 |  |  | 3.25 |
| Isovaleryl-CoA dehydrogenase IvdA | Afu5g08930 |  |  | 3.23 |
| Aspartate aminotransferase | Afu1g04160 |  |  | 3.22 |
| Zinc-dependent alcohol dehydrogenase | Afu1g14390 |  |  | 3.20 |
| MFS transporter | Afu1g01812 |  |  | 3.20 |
| Short chain dehydrogenase/reductase family oxidoreductase | Afu5g09290 |  |  | 2.98 |
| Oxidoreductase. short-chain dehydrogenase/reductase family | Afu5g14340 |  |  | 2.95 |
| Hypothetical protein | Afu3g11450 |  |  | 2.94 |
| Succinate dehydrogenase subunit Sdh1 | Afu3g07810 | *sdh1* |  | 2.90 |
| Oxidoreductase. 2-nitropropane dioxygenase family. putative | Afu2g09850 |  |  | 2.82 |
| Vacuolar carboxypeptidase Cps1 | Afu3g07040 | *cps1* |  | 2.82 |
| Hypothetical protein | Afu2g14450 |  |  | 2.73 |
| Sterol carrier protein | Afu4g06380 |  |  | 2.65 |

^a^This value represents the difference of the fold change in log_2_ obtained for each gene between days of infection compared in each case. Positive values indicated up-regulation relative to the first day post-infection. Data obtained with Agilent Whole *A. fumigatus* Genome Expression 44K v.1.

^b^Product description of the genes found on the microarray following RefSeq nomenclature.

^c^Systematic name of the gene following AspGD nomenclature.

^d^Gene name following AspGD nomenclature (<http://www.aspergillusgenome.org>), except fumagillin/pseurotin pathway that also follow the nomenclature published by Wieman P. *et al.* (2013).
