# Supplementary material for: A possible role for fumagillin in cellular damage during host infection by Aspergillus fumigatus
Source: Virulence. 2018 Sep 25;9(1):1548–61. doi: 10.1080/21505594.2018.1526528 (PMC6177242; doi:10.1080/21505594.2018.1526528)
Supplement: Supplemental Material [file kvir-09-01-1526528-s001.zip › Table S3_Primers.docx]

**Table S3.** Genes selected and *Aspergillus fumigatus* specific primers used in RT-qPCR assays to verificate the microarray data.

| **Target^a^** | **Symbol^b^** | **Primer**  **name^c^** | **Sequence (5´->3´)^d^** | **Tm^e^** | **%GC^f^** | **Amplicon (pb)^g^** |
| --- | --- | --- | --- | --- | --- | --- |
| Polyketide synthase (fma-PKS) | AFUA_8G00370 | 8g00370F  8g00370R | GCCATCGATTTCGATAACCT  GACGCTTGCACGTCTGTAGT | 59.00  59.12 | 45  55 | 90 |
| O-methyltransferase | AFUA_8G00390 | 8g00390F  8g00390R | CTTTGAGGAGGGAGTTGAGC  TGTCTCATCCGACCAGTCAT | 59.01  59.05 | 55  50 | 85 |
| Hypothetical protein | AFUA_8G00400 | 8g00400F  8g00400R | ATCGAAAAGCTCCAGACGTT  TCTTCTTTCGTTTCCAGCGT | 57.54  57.77 | 45  45 | 113 |
| Methionine aminopeptidase type II (metAP) | AFUA_8G00410 | 8g00410F  8g00410R | TTCCCGAACAACTCCTATCC  TTGTCGAGGTGACGTTTCTC | 58.98  58.85 | 50  50 | 95 |
| C6 transcription factor (fumR) | AFUA_8G00420 | 8g00420F  8g00420R | CCTCCTCGTTACAGGTCGAT  CGGTTGACTCGTATGGACAC | 59.16  59.01 | 55  55 | 80 |
| Hypothetical protein | AFUA_8G00430 | 8g00430F  8g00430R | GCCAAACTACATGCGAAGAA  TCCGACGTAAAGTCACGAAA | 58.92  59.32 | 45  45 | 80 |
| Monooxygenase | AFUA_8G00440 | 8g00440F  8g00440R | TCGAAAGGGTGATTGCCAAA  AGAGAGATACACGCGGAAGA | 58.01  57.96 | 45  50 | 143 |
| Phytanoil-CoA dioxygenase | AFUA_8G00480 | 8g00480F  8g00480R | TCTGGTTCCAACGTGACTGA  CATGTGAGGCGCCGAATAAT | 58.88  58.77 | 50  50 | 69 |
| Acetate CoA ligase | AFUA_8G00500 | 8g00500F  8g00500R | AGGGGTCAAGAAGGGTGATG  AACAGCTCCCAGACGATTGA | 59  59.02 | 55  50 | 94 |
| Cytochrome P450 oxidoreductase OrdA-like | AFUA_8G00510 | 8g00510F  8g00510R | CCCTGATCCGAGCGAAATTG  TCTCGTTGCCTTGGTCATCT | 59.06  59.02 | 55  50 | 146 |
| Alpha/beta hydrolase | AFUA_8G00530 | 8g00530F  8g00530R | ATCCTCGGCGTTCTCAAGAA  CAAGTATTCCGTGCCGTCTG | 59.1  59 | 50  55 | 147 |
| Defensin domain protein | AFUA_7G05180 | 7g05180F  7g05180R | TCCACCTGCAACAACGTCT  CATCAATCACCTCGTCGCTG | 59.48  59.07 | 52.63  55 | 133 |
| Extracelular lipase | AFUA_5G02040 | 5g02040F  5g02040R | GCAACATTCATCACCGGACA  TCTGGGCTAATGTGGCTGAA | 58.84  59.01 | 50  50 | 107 |
| Extracelular lipase | AFUA_7G04020 | 7g04020F  7g04020R | TGGCCATCTACCCGAAAAGT  ACAAGTGACCTCATGACCGT | 59.01  58.95 | 50  50 | 80 |
| Methionine aminopeptidase. type II | AFUA_2G01750 | 2g01750F  2g01750R | TTTTCTCGCCGGTATCTCGA  AACCCTTCACGTCAACAAGC | 58.90  58.98 | 50  55 | 79 |
| Secreted dipeptidyl peptidase DppV | AFUA_2G09030 | 2g09030F  2g09030R | CGTCAAATGCTGGGAGTACG  AATTGATCATGAACCCGCCG | 59  58.98 | 55  50 | 98 |
| Elastinolytic metalloproteinase Mep | AFUA_8G07080 | 8g07080F  8g07080R | ACCCTCTAACGTACACCAGC  AACACCTCGTACAGCATGGA | 59.11  59.03 | 55  50 | 85 |
| Allergen and cytotoxin AspF1 | AFUA_5G02330 | 5g02330F  5g02330R | CACGCCCATCAAATTCGGAA  TCCTTGGGTTTCTTCGAGTCA | 59.19  58.96 | 50  47.62 | 147 |
| Dipeptidyl-peptidase DppIV | AFUA_4G09320 | 4g09320F  4g09320R | CTACACCAAGCAATACCGGC  CGGTCGCAATTTCATCGACT | 58.99  59 | 55  50 | 76 |
| Vacuolar carboxypeptidase Cps1 | AFUA_3G07040 | 3g07040F  3g07040R | GGCAGCATCGATATTGTGCT  GATGATCTCGGCCATGATGC | 58.77  58.91 | 50  55 | 96 |
| C6 transcription factor | AFUA_5G14290 | 5g14290F  5g14290R | AACGTGCAAGCCCTGTTAAG  CCATGCATGAAGAAGGGACG | 59.05  58.98 | 50  55 | 50 |
| C6 sexual development transcription factor NosA | AFUA_4G09710 | 4g09710F  4g09710R | ACCTTTCAGCATGACCCTCA  CGATAGGTGTGGGCGAATTG | 58.93  59.06 | 50  55 | 87 |
| Class V chitinase ChiB1 | AFUA_8G01410 | 8g01410F  8g01410R | CGGGAGTCTTCGAACAGAGT  GCCATTGCGCAGGTTATCAT | 59.12  59.33 | 55  50 | 71 |
| C6 transcription factor | AFUA_5G00950 | 5g00950F  5g00950R | CCTCCTTTTCGTAGCCAAGC  TTGTCCAGATGATCGCCGTA | 58.91  58.89 | 55  50 | 56 |
| Pectin lyase | AFUA_5G10170 | 5g10170F  5g10170R | CGTCACCATCTCCAACAACG  AGTCCAGTAGTGCTTGCCAT | 59.21  59.01 | 55  50 | 76 |
| Pectin lyase | AFUA_5G10380 | 5g10380F  5g10380R | GAACAAATCCCTGATCGGCC  GGTTGATGTCCGTGATAGCG | 58.97  58.79 | 55  55 | 116 |
| Hypothetical protein | AFUA_5G08800 | 5g08800F  5g08800R | GTCATCAAACCGCCCTCATC  ACTTGTGGCAGGCAGATAGT | 58.98  59.01 | 55  50 | 139 |
| Hypothetical protein | AFUA_3G00410 | 3g00410F  3g00410R | ACGTCTCCTTGGAGCAAAGA  TATACTGTTCGCGGGCATCT | 58.95  58.96 | 50  50 | 140 |
| Ankyrin repeat protein | AFUA_7G08610 | 7g08610F  7g08610R | CAGCAAAGGTCCGATTCAGG  TTCAGCCGTAAAAGTTCGCC | 58.91  59.13 | 55  50 | 86 |
| Aryl-alcohol oxidase; vanillyl-alcohol oxidase | AFUA_3G09500 | 3g09500F  3g09500R | CTTTGGCCCTGTTATCGAGC  ATCCGCTTCTTCCCACTTGA | 58.98  59.02 | 55  50 | 96 |
| Acetyltransferase. GNAT family | AFUA_1G09260 | 1g09260F  1g09260R | ACTGTGGTCTCCTCGTCTTG  TGTGCGAGGATGTCAAATGC | 59.04  59.20 | 55  50 | 87 |
| Short chain dehydrogenase/oxidoreductase CpoX2 | AFUA_2G18000 | 2g18000F  2g18000R | GCCTGCGCCTATTTCACTAC  ATTGGCGTGTTTGTGACTCC | 59.06  59.05 | 55  50 | 95 |
| Xylosidase/glycosyl hydrolase | AFUA_2G00930 | 2g00930F  2g00930R | GGGGCTCCGTATACACTGAA  CCCAGCGAGACAGTAGCATA | 58.88  58.97 | 55  55 | 86 |
| Aldehyde dehydrogenase | AFUA_7G01000 | 7g01000F  7g01000R | CTATGACAAGTTCCTGGCGC  ATCCGGTCAAATTGCACCTG | 58.99  58.82 | 55  50 | 117 |
| Maleylacetoacetate isomerase MaiA | AFUA_2G04240 | 2g04240F  2g04240R | AATCCTTCCGGTACAGTCCC  GATGGTCACGGGTTCTTTGG | 58.80  58.83 | 55  55 | 63 |
| 4-Hydroxyphenylpyruvate dioxygenase | AFUA_2G04200 | 2g04200F  2g04200R | TATGCAGCGACGAACAATGG  CTAAAGTGTGCGTGGTCTCG | 58.99  58.94 | 50  55 | 118 |
| Indoleamine 2.3-dioxygenase family protein | AFUA_7G02010 | 7g02010F  7g02010R | GCCGCTTATTCGCTCTTCAA  GTTGTCGTAAACCAAGCCCT | 58.99  58.40 | 50  50 | 63 |
| Methyltransferase SirN-like | AFUA_8G00550 | 8g00550F  8g00550R | ACGTTGAAGGCGAGTTGTTC  ATGGGCGAAATAGGTGATGC | 59.06  58.39 | 50  50 | 56 |
| Glutathione S-transferase elfB | AFUA_8G00580 | 8g00580F  8g00580R | CGACCAGTGCCGAACAATAG  TTCGCGAATATCCAGGTCGA | 59  58.97 | 55  50 | 70 |
| Hypothetical protein | AFUA_1G10450 | 1g10450F  1g10145R | AGGGACCAGATGACGAGTTG  ATTCCCTTCCATGAGCGTGA | 59.10  59.09 | 55  50 | 98 |
| Hypothetical protein | AFUA_2G16440 | 2g16440F  2g16440R | GTTGGGCCAATGTATCGACC  GAGGAAGCCATGTCCGTCTA | 58.98  58.89 | 55  55 | 132 |
| Integral membrane protein Pth11-like | AFUA_7G06620 | 7g06620F  7g06620R | TCATCGGCGAGTACTCATCC  ACTGTCTTTGAAGCGGGAGA | 59.05  58.95 | 55  50 | 95 |
| MFS sugar transporter | AFUA_3G03700 | 3g03700F  3g03700R | TCAGTTTAGTTGCTCCCATTGC  ACCGATTCCCAACAGTATACGA | 59.18  58.97 | 45.45  45.45 | 83 |
| *fmaA* complementation | AFUA_8g00520 | fmacomhygF  fmacomhygR | GGCATCTTTAAGCTTGAACCAGTGCAGATACTCGG  GATCTACGAAAGCTTGACGAGTCTCCAAGATGGCG | 64.10  64.70 | 48.57  51.43 | 3,036 |
| **Reference genes** | | | | | | |
| Alpha/beta hydrolase | AFUA_2G02920 | H_AlphBet_F  H_AlphBet_R | ACTGGTCGTGACACTGTTGG  CGCATTGTGGATGCTAGACT | 59.62  58.90 | 55  50 | 102 |
| Mis12-Mtw1 family protein | AFUA_3G13950 | H_Mis12_F  H_Mis12_R | GCTCTCGAATTGGTTTGGAC  ATGTTTTTCGGGTTCGGTTT | 59.68  60.57 | 50  40 | 75 |
| Isochorismatase family protein | AFUA_3G14500 | H_Isochor_F  H_Isochor_R | GAAGAACGACGGAGTGGTAAG  CTCAGGTAGGTCCAAGTCATAAAT | 62  62 | 52.4  41.7 | 108 |
| Molybdopterin synthase small subunit CnxG | AFUA_7G01580 | H_Molybdop_F  H_Molybdop_R | ACCAGTCTTCCAAATCCACTAC  TAGGAAATCAAAGAGCCTGGAC | 62  62 | 45.5  45.5 | 106 |
| **Mus musculus reference gene** | | | | | | |
| Glucose-6-phosphate 1-dehydrogenase 2 | NM_019468.2 | MmF  MmR | CCTTTGGTACTGAGGGTCGT  ATCCATTGGCAGCTTCTCCT | 59.03  59.08 | 55  50 | 246 |

^a^Product description of the genes chosen to verificate the microarray data following RefSeq nomenclature.

^b^Systematic name of the gene following AspGD nomenclature.

^c^F: Forward; R: Reverse

^d^Sequence of each primer.

^e^Tm: Melting temperature of each primer.

^f^%GC: Percentage of guanine and cytosine content of each primer.

^g^Size of cDNA amplification product.
