# Supplementary material for: A possible role for fumagillin in cellular damage during host infection by Aspergillus fumigatus
Source: Virulence. 2018 Sep 25;9(1):1548–61. doi: 10.1080/21505594.2018.1526528 (PMC6177242; doi:10.1080/21505594.2018.1526528)
Supplement: Supplemental Material [file kvir-09-01-1526528-s001.zip › Table S4_GO.docx]

| **DEGs Down regulated** |  |  |  |  |
| --- | --- | --- | --- | --- |
| **Cellular Component** |  |  |  |  |
| **GOID** | **GO term** | **Frequency** | **Percentage (%)** | **Gene(s)** |
| 5575 | Unknown | 16 out of 18 genes | 88,89% | Afu1g09260 Afu3g09500 Afu2g16440 Afu8g01795 Afu3g14390 Afu7g08610 Afu3g00410 Afu2g1800 Afu4g08310 Afu5g14850  Afu1g12475 Afu7g07140 Afu1g10450 Afu6g03330 Afu5g10170 Afu8g06300 |
| 5576 | Extracellular region | 1 out of 18 genes | 5,56% | Afu5g10380 |
| 5634 | Nucleus | 1 out of 18 genes | 5,56% | Afu5g00950 |
|  |  |  |  |  |
| **Molecular Function** |  |  |  |  |
| **GOID** | **GO term** | **Frequency** | **Percentage (%)** | **Gene(s)** |
| 3674 | Unknown | 13 out of 18 genes | 72,22% | Afu2g16440 Afu8g01795 Afu3g14390 Afu7g08610 Afu3g00410  Afu4g08310 Afu5g14850 Afu1g12475 Afu7g07140 Afu1g10450  Afu6g03330 Afu5g10170 Afu8g06300 |
| 16491 | Oxidoreductase activity | 2 out of 18 genes | 11,11% | Afu3g09500 Afu2g1800 |
| 16829 | Lyase activity | 1 out of 18 genes | 5,56% | Afu5g10380 |
| 16740 | Transferase activity | 1 out of 18 genes | 5,56% | Afu1g09260 |
| other | Other | 1 out of 18 genes | 5,56% | Afu5g00950 |
|  |  |  |  |  |
| **Biological Process** |  |  |  |  |
| **GOID** | **GO term** | **Frequency** | **Percentage (%)** | **Gene(s)** |
| 8150 | Unknown | 14 out of 18 genes | 77,78% | Afu1g09260 Afu2g16440 Afu8g01795 Afu3g14390 Afu7g08610  Afu3g00410 Afu4g08310 Afu5g14850 Afu1g12475 Afu7g07140  Afu1g10450 Afu6g03330 Afu5g10170 Afu8g06300 |
| 50789 | Regulation of biological process | 1 out of 18 genes | 5,56% | Afu5g00950 |
| 5975 | Carbohydrate metabolic process | 1 out of 18 genes | 5,56% | Afu5g10380 |
| other | Other | 2 out of 18 genes | 11,11% | Afu3g09500 Afu2g1800 |

**Table S4.** Results of DEGs GO enrichment.

| **DEGs Up regulated** |  |  |  |  |
| --- | --- | --- | --- | --- |
| **Cellular Component** |  |  |  |  |
| **GOID** | **GO term** | **Frequency** | **Percentage (%)** | **Gene(s)** |
| 5575 | Unknown | 57 out of 85 genes | 67,05% | Afu3g11450 Afu6g02210 Afu5g14340 Afu1g15160 Afu7g01930 Afu8g00430 Afu4g02790 Afu5g08930 Afu8g00400 Afu1g02290 Afu4g10610 Afu8g00480 Afu5g02330 Afu1g09030 Afu8g00510 Afu7g06620 Afu1g04160 Afu8g00910 Afu8g00410 Afu1g15350 Afu3g13080 Afu8g00500 Afu2g09030 Afu3g08610 Afu1g14390 Afu5g02320 Afu5g00550 Afu1g04130 Afu5g08800 Afu8g00370 Afu5g00590 Afu5g00700 Afu2g04200 Afu8g00440 Afu5g09290 Afu5g02040 Afu2g01750 Afu8g00560 Afu6g10130 Afu6g13740 Afu7g02010 Afu7g06130 Afu4g12700 Afu7g04020 Afu7g05180 Afu8g00710 Afu8g00390 Afu2g04380 Afu2g08820 Afu6g08470 Afu1g00450 Afu2g14450 Afu8g00530 Afu7g06140 Afu5g02670 Afu6g01870 Afu6g00430 |
| 16020 | Membrane | 8 out of 85 genes | 9,41% | Afu4g0930 Afu4g01560 Afu3g07050 Afu3g07810 Afu1g00440 Afu2g17840 Afu6g13190 Afu3g03700 |
| 5576 | Extracellular region | 8 out of 85 genes | 9,41% | Afu4g0930 Afu6g04920 Afu1g14190 Afu2g17630 Afu5g10930 Afu8g07080 Afu8g01410 Afu7g01000 |
| 5739 | Mitochondrion | 5 out of 85 genes | 5,88% | Afu5g08940 Afu6g12250 Afu3g07810 Afu8g05530 Afu7g01000 |
| 5829 | Cytosol | 4 out of 85 genes | 4,71% | Afu6g04920 Afu8g00580 Afu8g05530 Afu2g06000 |
| 5634 | Nucleus | 4 out of 85 genes | 4,71% | Afu5g14290 Afu8g05530 Afu8g00420 Afu4g09710 |
| 5777 | Peroxisome | 3 out of 85 genes | 3,52% | Afu8g05530 Afu4g06380 Afu2g09850 |
| 5618 | Cell wall | 1 out of 85 genes | 0,85% | Afu3g07050 |
| 5773 | Vacuole | 1 out of 85 genes | 0,85% | Afu3g07040 |
| 5886 | Plasma membrane | 1 out of 85 genes | 0,85% | Afu6g13190 |
| Other | Other | 4 out of 85 genes | 4,71% | Afu6g03590 Afu2g10230 Afu8g00550 Afu2g04240 |
| **Molecular Function** |  |  |  |  |
| **GOID** | **GO term** | **Frequency** | **Percentage (%)** | **Gene(s)** |
| 3674 | Unknown | 39 out of 85 genes | 45,88% | Afu3g11450 Afu3g07050 Afu1g15160 Afu7g01930 Afu8g00430 Afu8g00400 Afu4g10610 Afu8g00480 Afu1g14190 Afu1g09030 Afu7g06620 Afu8g00910 Afu1g15350 Afu3g13080 Afu2g17630 Afu3g08610 Afu5g02320 Afu1g00440 Afu5g10930 Afu1g04130 Afu5g08800 Afu8g00580 Afu5g00590 Afu5g00700 Afu2g17840 Afu6g13740 Afu7g06130 Afu4g12700 Afu7g04020 Afu7g05180 Afu8g00710 Afu2g04380 Afu2g08820 Afu2g14450 Afu8g00530 Afu5g02670 Afu6g01870 Afu8g00550 Afu6g00430 |
| 16491 | Oxidoreductase activity | 17 out of 85 genes | 20% | Afu6g02210 Afu5g14340 Afu6g04920 Afu5g08930 Afu3g07810 Afu8g00510 Afu1g14390 Afu8g00370 Afu2g04200 Afu8g00440 Afu5g09290 Afu8g00560 Afu8g05530 Afu2g10230 Afu7g01000 Afu2g09850 Afu2g06000 |
| 16787 | Hydrolase activity | 14 out of 85 genes | 16,47% | Afu4g0930 Afu4g02790 Afu5g02330 Afu8g00410 Afu2g09030 Afu5g00550 Afu5g02040 Afu2g01750 Afu6g10130 Afu8g07080 Afu8g01410 Afu3g07040 Afu1g00450 Afu7g06140 |
| 16740 | Transferase activity | 9 out of 85 genes | 15,59% | Afu6g12250 Afu1g04160 Afu1g14390 Afu8g00370 Afu6g03590 Afu4g06380 Afu8g01410 Afu8g00390 Afu6g08470 |
| 8233 | Peptidase activity | 6 out of 85 genes | 7,05% | Afu4g0930 Afu8g00410 Afu2g09030 Afu2g01750 Afu8g07080 Afu3g07040 |
| 5215 | Transporter activity | 3 out of 85 genes | 3,52% | Afu4g01560 Afu6g13190 Afu3g03700 |
| 16298 | Lipase activity | 1 out of 85 genes | 0,85% | Afu5g02040 |
| 5515 | Protein binding | 1 out of 85 genes | 0,85% | Afu5g02330 |
| 16853 | Isomerase activity | 1 out of 85 genes | 0,85% | Afu2g04240 |
| 3677 | DNA binding | 1 out of 85 genes | 0,85% | Afu5g14290 |
| 3723 | RNA binding | 1 out of 85 genes | 0,85% | Afu5g02330 |
| 16874 | Ligase activity | 1 out of 85 genes | 0,85% | Afu5g08940 |
| Other | Other | 5 out of 85 genes | 5,88% | Afu1g02290 Afu8g00500 Afu7g02010 Afu8g00420 Afu4g09710 |
| **Biological Process** |  |  |  |  |
| **GOID** | **GO term** | **Frequency** | **Percentage (%)** | **Gene(s)** |
| 8150 | Unknown | 31 out of 85 genes | 36,47% | Afu3g11450 Afu1g15160 Afu1g02290 Afu4g10610 Afu1g14190 Afu1g09030 Afu7g06620 Afu1g15350 Afu3g13080 Afu2g17630 Afu3g08610 Afu5g02320 Afu5g10930 Afu1g04130 Afu8g00580 Afu5g00590 Afu5g00700 Afu8g07080 Afu6g13740 Afu7g02010 Afu7g06130 Afu4g12700 Afu7g04020 Afu2g04380 Afu2g08820 Afu2g14450 Afu8g00530 Afu5g02670 Afu6g01870 Afu8g00550 Afu6g00430 |
| 19748 | Secondary metabolic process | 13 out of 85 genes | 15,29% | Afu8g00430 Afu8g00400 Afu8g00480 Afu8g00510 Afu8g00910 Afu8g00410 Afu8g00500 Afu8g00370 Afu2g04200 Afu6g03590 Afu8g00440 Afu8g00420 Afu8g00390 |
| 50789 | Regulation of biological process | 9 out of 85 genes | 15,59% | Afu3g07050 Afu7g01930 Afu5g02330 Afu5g14290 Afu5g08800 Afu8g00420 Afu8g00710 Afu7g01000 Afu4g09710 |
| 5975 | Carbohydrate metabolic process | 7 out of 85 genes | 8,24% | Afu5g00550 Afu6g03590 Afu6g10130 Afu8g01410 Afu2g10230 Afu6g08470 Afu7g06140 |
| 6520 | Cellular amino acid metabolic process | 5 out of 85 genes | 5,88% | Afu5g08940 Afu1g04160 Afu7g01000 Afu2g06000 Afu2g04240 |
| 6810 | Transport | 5 out of 85 genes | 5,88% | Afu4g01560 Afu1g00440 Afu2g17840 Afu6g13190 Afu3g03700 |
| 6629 | Lipid metabolic process | 4 out of 85 genes | 4,71% | Afu8g00370 Afu6g03590 Afu5g02040 Afu4g06380 |
| 32502 | Developmental process | 4 out of 85 genes | 4,71% | Afu7g01930 Afu5g08800 Afu8g01410 Afu4g09710 |
| 42221 | Response to chemical | 3 out of 85 genes | 3,52% | Afu2g10230 Afu6g13190 Afu7g01000 |
| 6950 | Response to stress | 3 out of 85 genes | 3,52% | Afu5g08800 Afu7g05180 Afu8g00710 |
| 30163 | Protein catabolic process | 2 out of 85 genes | 2,35% | Afu4g0930 Afu3g07040 |
| 16070 | RNA metabolic process | 2 out of 85 genes | 2,35% | Afu5g14290 Afu4g09710 |
| 34293 | Sexual sporulation | 2 out of 85 genes | 2,35% | Afu7g01930 Afu4g09710 |
| 9404 | Toxin metabolic process | 2 out of 85 genes | 2,35% | Afu8g00370 Afu6g03590 |
| 30447 | Filamentous growth | 2 out of 85 genes | 2,35% | Afu5g08800 Afu4g09710 |
| 7049 | Cell cycle | 2 out of 85 genes | 2,35% | Afu7g01930 Afu4g09710 |
| 30436 | Asexual sporulation | 2 out of 85 genes | 2,35% | Afu7g01930 Afu4g09710 |
| 6351 | Transcription, DNA-templated | 2 out of 85 genes | 2,35% | Afu5g14290 Afu4g09710 |
| 45333 | Cellular respiration | 1 out of 85 genes | 0,85% | Afu3g07810 |
| 55085 | Transmembrane transport | 1 out of 85 genes | 0,85% | Afu1g01812 |
| Other | Other | 14 out of 85 genes | 16,47% | Afu6g02210 Afu5g14340 Afu6g04920 Afu4g02790 Afu5g08930 Afu6g12250 Afu2g09030 Afu1g14390 Afu5g09290 Afu2g01750 Afu8g00560 Afu8g05530 Afu2g09850 Afu1g00450 |
