# Supplementary figures and images for: A possible role for fumagillin in cellular damage during host infection by Aspergillus fumigatus
Source: Virulence. 2018 Sep 25;9(1):1548–61. doi: 10.1080/21505594.2018.1526528 (PMC6177242; doi:10.1080/21505594.2018.1526528)

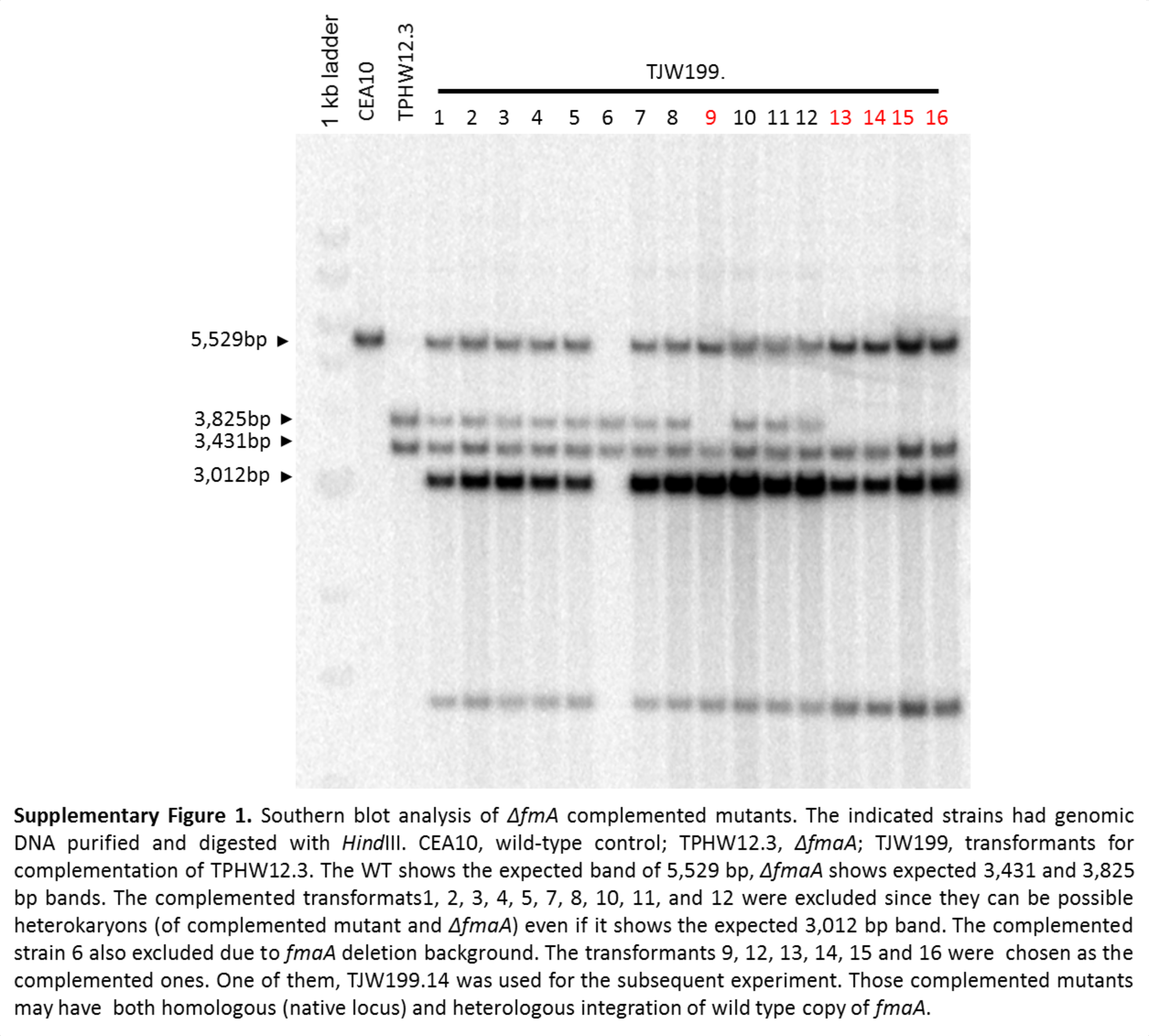

Supplement: Supplemental Material [file kvir-09-01-1526528-s001.zip › FigS1.tif]
